# Impact of Metformin Use on Clinical and Pathological Outcomes in Breast Cancer Patients With Type 2 Diabetes

**DOI:** 10.1002/cnr2.70631

**Published:** 2026-07-29

**Authors:** Ensiyeh Bahadoran, Monir Mirzadeh, Mohsen Jafari, Yazdan Zafari, Sahar Moghbelinejad

**Affiliations:** ^1^ Cellular and Molecular Research Center, Research Institute for Prevention of Non‐Communicable Diseases Qazvin University of Medical Sciences Qazvin Iran; ^2^ Non‐Communicable Diseases Research Center, Research Institute for Prevention of Non‐Communicable Diseases Qazvin University of Medical Sciences Qazvin Iran; ^3^ School of Medicine, Qazvin University of Medical Sciences Qazvin Iran; ^4^ Department of Hematology and Medical Oncology, School of Medicine Qazvin University of Medical Sciences Qazvin Iran

**Keywords:** breast cancer, lymphatic metastasis, metformin, prognosis, type 2 diabetes mellitus

## Abstract

**Background:**

Breast cancer is the most common malignancy in women, and its incidence is increasing worldwide. Type 2 diabetes mellitus (T2DM) is a significant comorbidity in patients with breast cancer that negatively affects prognosis. Metformin, a first‐line antidiabetic drug, has been suggested to possess antineoplastic properties. This study investigated the effects of metformin on the demographic characteristics, pathological parameters, treatment response, and prognosis of breast cancer patients with T2DM in an Iranian population.

**Methods:**

A retrospective cross‐sectional study was conducted on 104 patients with breast cancer and T2DM at the Velayat Educational and Therapeutic Center, Qazvin, Iran, from 2019 to 2023. The patients were categorized into two groups: metformin users (*n* = 46) and non‐users (*n* = 58). Data on demographic characteristics, disease stage, lymph node involvement, hormone receptor status, treatment response, prognosis, recurrence, and metastasis were collected. Statistical analyses were performed using SPSS version 25, with significance set at *p* < 0.05. Unadjusted and adjusted logistic regression analyses were performed to identify independent predictors of prognosis.

**Results:**

The mean disease duration was significantly shorter in the metformin group than in the non‐metformin group (*p* = 0.036). A significant difference was observed in the stage of disease (*p* = 0.003), with metformin users more frequently presenting at earlier stages. Lymph node involvement was significantly lower in the metformin group (*p* = 0.008). Patients using metformin showed significantly better treatment response (*p* = 0.028) and prognosis (*p* = 0.009). However, no significant differences were observed in the estrogen receptor (ER) status (*p* = 0.293), human epidermal growth factor receptor 2 (HER2) status (*p* = 0.762), metastasis (*p* = 0.249), or recurrence rates (*p* = 0.770). After adjustment, disease duration, HER2 status, and metformin use remained independently associated with prognosis.

**Conclusion:**

Metformin use in breast cancer patients with T2DM is associated with lower disease stage, fewer involved lymph nodes, improved treatment response, and better prognosis. These findings suggest that metformin may play a beneficial role in breast cancer progression and treatment outcomes. Further prospective studies are warranted to confirm these results in a clinical setting and to elucidate the underlying mechanisms.

## 1 Introduction

Breast cancer accounted for an estimated 2.3 million new cases in 2022, making it the second most common cancer globally (11.6% of all cases) and the fourth leading cause of cancer‐related deaths, responsible for 666 000 deaths worldwide. Breast cancer is the most frequent cancer among women, and it is the primary cause of cancer‐related deaths worldwide, occurring in 157 nations for incidence and 112 countries for mortality [1]. The mortality rates for breast cancer in young women have markedly decreased in high‐income nations, while they have risen in lower‐income countries. This gap highlights the consequences of inadequate investment in prevention strategies, health education, early detection, and treatment on the mortality of young women in lower‐income nations [2]. Breast cancer is expected to cause more than 3 million new cases and 1 million deaths annually by 2040 due to population growth and aging alone [3]. While the worldwide indicators for female breast cancer burden have stayed relatively constant, Iran has seen a nearly twofold increase in both incidence and prevalence, along with significant increases in mortality and adjusted years of life lost due to disabilities (DALYs) [4].

Diabetes is a metabolic condition characterized by elevated blood sugar levels due to deficiencies in either the action or secretion of insulin, or both. Type 2 diabetes mellitus (T2DM) is becoming one of the most common human diseases and the sixth most common cause of mortality worldwide. Its incidence is rapidly increasing [5]. Diabetes‐related chronic hyperglycemia is linked to permanent damage, malfunction, and failure of various organs, particularly the heart, blood vessels, kidneys, eyes, and nerves [6]. According to the International Diabetes Federation (IDF), an estimated 537 million adults aged 20–79 years were living with diabetes worldwide in 2021, representing 10.5% of the global population in this age group. This number is projected to increase to 643 million by 2030 and 783 million by 2045, highlighting the growing public health burden of diabetes [7]. Diabetes affects up to 16% of breast cancer patients [8]. The risk of both breast cancer‐specific and all‐cause death following breast cancer diagnosis is greater in women with diabetes mellitus [9]. In Iran, T2DM has become highly prevalent and continues to impose a considerable burden on the healthcare system. According to the Global Burden of Disease study, the number of T2DM cases increased from 1990 to 2019, reaching 5 035 012 cases in 2019 (a 417% increase), while incidence cases reached 291 482 in 2019 (a 374% increase) [10]. As diabetes is a common comorbidity among breast cancer patients [11], investigating the potential impact of metformin on breast cancer outcomes in Iranian women is of particular clinical relevance.

Metformin is the first‐line treatment for T2DM, especially in patients who are overweight or obese. Research indicates that metformin may have anticancer properties, and observational studies suggest that using metformin lowers the overall incidence of breast cancer in patients with diabetes [12]. There are several mechanisms underlying metformin's antiproliferative actions [13]. Analysis of breast cancer cell lines has shown that metformin inhibits growth by upregulating the activity of AMP‐activated protein kinase (AMPK) and consequently suppressing signaling via the mammalian target of rapamycin (mTOR) [14]. Additional pathways include the inhibition of human epidermal growth factor receptor‐2 (HER2) protein expression and effects on estrogen production and estrogenic signal transmission [15].

Many studies have explored the association between T2DM and breast cancer. For example, meta‐analyses report a ~20%–30% increased breast cancer risk in women with T2DM [16], and cohort studies confirm worse breast‐cancer survival in diabetic patients [17]. However, most available evidence regarding the impact of metformin on breast cancer outcomes originates from North American, European, and East Asian populations [16], with limited data from Middle Eastern regions, including Iran. Therefore, the present study provides population‐specific, real‐world clinical evidence by evaluating the association between metformin use and clinicopathological characteristics and outcomes in Iranian women with concurrent T2DM and breast cancer.

## 2 Methods

This retrospective cross‐sectional study was conducted at the Velayat Educational and Therapeutic Center affiliated with Qazvin University of Medical Sciences, Qazvin, Iran. This study was conducted between 2019 and 2023. The Ethics Committee of the Qazvin University of Medical Sciences, Qazvin, Iran (IR.QUMS.REC.1400.143) approved the study protocol. Written informed consent was obtained from all participants. All the patient data were de‐identified to ensure confidentiality. The present study was performed in accordance with relevant guidelines and regulations. The study population included patients who were referred to the oncology department of Velayat Center and had a concurrent diagnosis of breast cancer and T2DM. The inclusion criteria were a confirmed diagnosis of breast cancer, diabetes, and complete medical records available for review. Patients were treated with metformin at standard doses (500–2000 mg/day). The exclusion criteria were incomplete medical records, unconfirmed diabetes diagnosis, male sex, the presence of other malignancies, bilateral tumors, and distant metastases. Moreover, a group of patients was simultaneously treated with other types of hypoglycemic agents, such as sulfonylureas (gliclazide, glimepiride), glinides (repaglinide), and α‐glucosidase inhibitors (acarbose), was excluded to minimize treatment heterogeneity and allow a more focused comparison between metformin users and non‐users. Patients in the non‐metformin group included individuals who were managed with insulin therapy alone, lifestyle modification (diet and physical activity), or who had contraindications or intolerance to metformin. Therefore, these patients were excluded from this study. Finally, a total of 104 patients were enrolled in the final analysis, including 46 metformin users and 58 non‐users. Patients who fulfilled one of the following criteria were categorized as having diabetes: fasting plasma glucose level of ≥ 126 mg/dL (≥ 7.0 mmol/L); 2‐h value of ≥ 200 mg/dL (≥ 11.1 mmol/L) in the 75 g oral glucose tolerance test (OGTT); casual plasma glucose level of ≥ 200 mg/dL (≥ 11.1 mmol/L); or HbA1c ≥ 6.5% were regarded as indicators of diabetes [18].

Data were collected using a convenience sampling method. They included demographic characteristics (age and family history of breast cancer), pathological data (stage of the disease, lymph node involvement, and ER and HER2 receptors), and treatment information (use of metformin, treatment response, disease prognosis, metastasis, and recurrence).

Primary tumor staging was based on the pathological cancer staging classification developed by the American Joint Committee on Cancer. We employed clinical staging based on imaging investigations and clinical assessments if pathological tumor size was absent. The maximum tumor size was used to determine the tumor stage (Tis = ductal carcinoma in situ, T1a = ≤ 0.5 cm (including micro‐invasion), T1b = > 0.5 cm and ≤ 1 cm, T1c = > 1 cm and ≤ 2 cm, T2 = > 2 cm and ≤ 5 cm, T3 = > 5 cm, T4 = any size with direct extension to chest wall and/or skin) [19]. The number of regional lymph nodes with pathologically confirmed metastases was used to characterize lymph node status. Sentinel lymph node biopsy findings were used to determine whether the lymph nodes were positive [20]. Pathology, imaging, and clinical evaluation are used to evaluate treatment response [21].

According to the American Society of Clinical Oncology and College of American Pathologists, immunohistochemistry (IHC) is used to identify estrogen receptor (ER) status in breast cancer (BC), with nuclear expression in ≥ 1% of cells classified as ER‐positive [22]. When HER2 protein expression was IHC 3+ positive, it was considered HER2‐positive. If HER2 was 2+ positive on IHC, immunofluorescence hybridization (FISH) was performed for HER2. If the average HER2 gene copy number was 6.0 signals/cell or the HER2/CEP17 ratio was 2.0, then FISH was positive [23]. For this study, prognosis was defined based on the patient's clinical status recorded in the medical records at the most recent follow‐up. Patients were classified as having a favorable clinical outcome if they remained disease‐free and/or demonstrated a favorable treatment response. This definition was used as a clinical outcome measure and does not represent established survival endpoints such as overall survival (OS), disease‐free survival (DFS), or progression‐free survival (PFS). Recurrence describes the occurrence of tumors on the same side of the chest wall or in nearby lymph nodes that have been verified by pathology following radical surgery. When postoperative pathology or imaging shows distant organ metastases, such as those to the liver, lung, or bone, it is referred to as distant metastasis [24]. Disease duration was defined as the length of time since the breast cancer diagnosis. Treatment response was defined as a qualitative variable based on clinical assessment and categorized as good or weak. A good treatment response indicated an adequate and favorable response to therapy during the treatment period, whereas a weak treatment response reflected an insufficient or poor response.

Statistical analyses were performed using SPSS software version 25. Continuous variables were reported as means and standard deviations, while categorical variables were expressed as frequencies and percentages. The independent *t*‐test was used to examine the relationships between continuous and binary categorical variables, whereas the chi‐square test was used to assess the associations between categorical variables. A *p*‐value < 0.05 was considered statistically significant.

To identify factors independently associated with prognosis, unadjusted and adjusted logistic regression analyses were performed. The final model included duration of disease, lymph node involvement, HER2 status, and metformin use. Regression coefficients (B), odds ratios (Exp[B]), 95% confidence intervals (95% CI), and *p*‐values were reported. A two‐sided *p*‐value < 0.05 was considered statistically significant.

## 3 Results

This study evaluated 104 diabetic patients with breast cancer, of whom 46 were using metformin and 58 were not. The results are presented in the following section (Table 1).

**TABLE 1:** Comparison of clinical and pathological characteristics between diabetic breast cancer patients using and not using metformin.

Variable	Metformin users (*n* = 46)	Non‐metformin users (*n* = 58)	*p*
Age (years) (mean ± SD)	54.11 ± 11.34	54.47 ± 10.94	0.871
Disease duration (years) (median (IQR))	6 (4–8.25)	7.5 (5–12)	0.036
Positive family history (%)	46.2% (30)	53.8% (35)	0.685
Stage 0 (%)	100% (1)	0% (0)	0.003
Stage 1–2 (%)	57.8% (37)	42.1% (27)	
Stage 3–4 (%)	20.5% (8)	79.4% (31)	
Lymph node involvement (%)	34.3% (23)	65.7% (44)	0.008
Positive treatment response (%)	54.2% (32)	45.8% (27)	0.028
ER positive (%)	46.3% (44)	53.7% (51)	0.293
HER2 positive (%)	50% (6)	50% (6)	0.762
Good prognosis (%)	55.9% (33)	44.1% (26)	0.009
Metastasis (%)	33.3% (8)	66.7% (16)	0.249
Recurrence (%)	38.5% (5)	61.5% (8)	0.770

*Note:* Values are presented as mean ± standard deviation for normally distributed variables and median (interquartile range) for non‐normally distributed variables.

### 3.1 Demographic Characteristics

The mean age of patients in the metformin and non‐metformin group was 54.11 ± 11.34 and 54.47 ± 10.94 years, respectively. There was no statistically significant difference in age between the two groups (*p* = 0.871). The duration of the disease in the metformin and non‐metformin group was 7.04 ± 3.93 and 9 ± 5.16 years, respectively. This showed a significantly shorter duration of disease in the metformin group (*p* = 0.036).

### 3.2 Family History of Breast Cancer

In the metformin group, 30 patients had a family history of breast cancer, whereas in the non‐metformin group, 35 patients had a family history of breast cancer. There were no significant differences between the two groups regarding family history of breast cancer (*p* = 0.685).

### 3.3 Disease Stage

**FIGURE 1:**
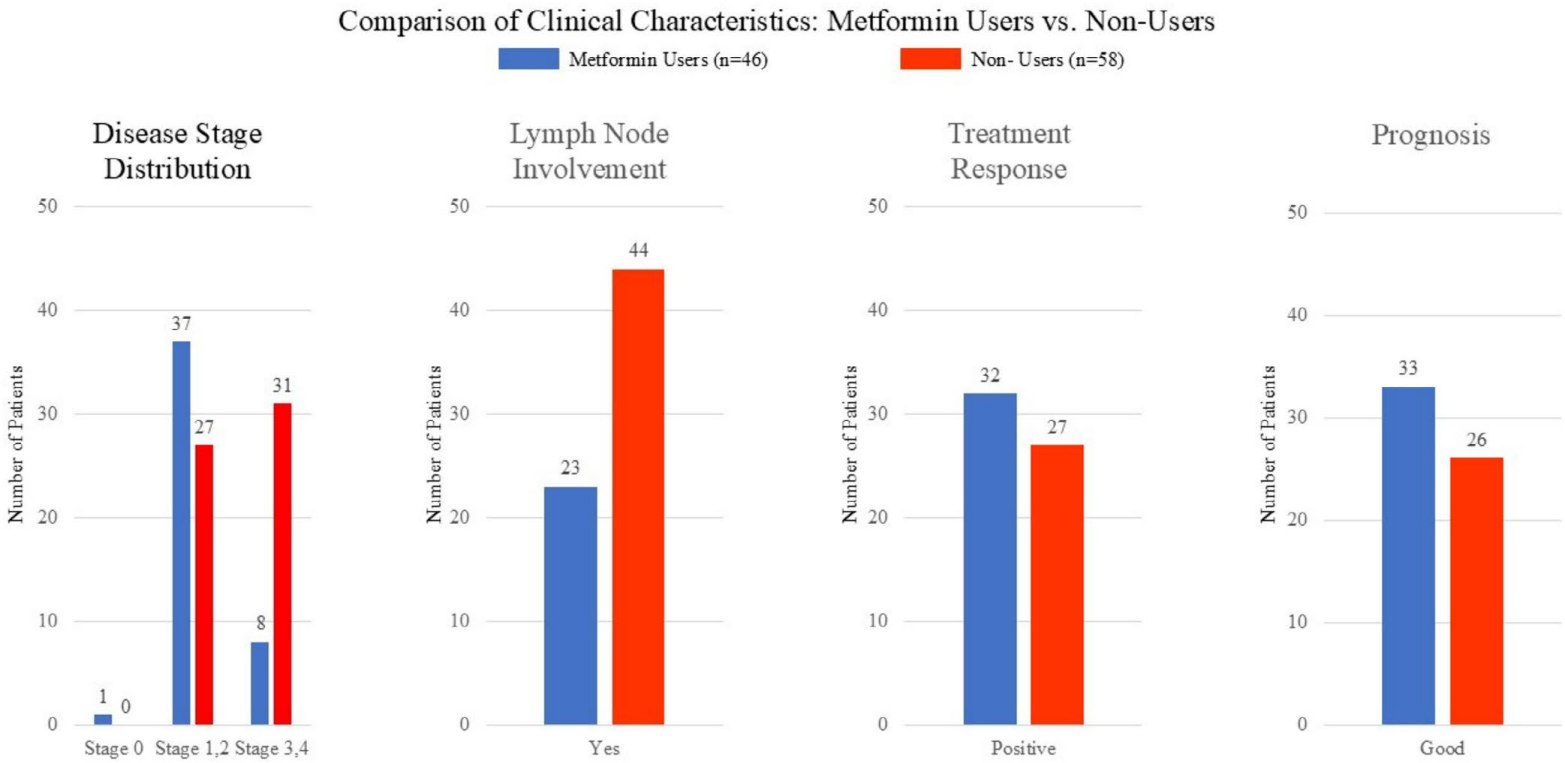
Comparison of clinicopathological characteristics between metformin users and non‐metformin users. Distribution of disease stage, lymph node involvement, treatment response, and prognosis among diabetic breast cancer patients according to metformin use. The metformin group showed a higher proportion of early‐stage disease, lower lymph node involvement, better treatment response, and more favorable prognosis compared with the non‐metformin group.

Early‐stage disease (stage 1–2) was more common among metformin users (*n* = 37), whereas advanced‐stage disease (stage 3–4) was more frequent among non‐metformin users (*n* = 31). This shows a significant difference in the disease stage between the groups (*p* = 0.003). It can be said that patients in the metformin group were more frequently at earlier stages of the disease, whereas those in the non‐metformin group were more often at an advanced stage of the disease (Figure 1).

### 3.4 Lymph Node Involvement

Patients in the metformin group had significantly fewer involved lymph nodes compared to the non‐metformin group (*p* = 0.008). Lymph node involvement was observed in 23 metformin users and 44 non‐metformin users (Figure 1).

### 3.5 Treatment Response

The metformin group demonstrated a significantly better response to treatment compared to the non‐metformin group (*p* = 0.028). A positive treatment response was observed in 32 metformin users and 27 non‐metformin users (Figure 1).

### 3.6 Hormone Receptor Status

No significant differences were observed between the groups in terms of ER (*p* = 0.293) or HER2 (*p* = 0.762) receptor positivity.

### 3.7 Cancer Prognosis and Metastasis and Recurrence

A favorable prognosis was observed in 33 metformin users and 26 non‐metformin users, and the difference was statistically significant (*p* = 0.009). However, there was no significant difference between the groups in terms of metastasis (*p* = 0.249) or disease recurrence (*p* = 0.770) (Figure 1).

Overall, Figure 1 visually summarizes the significant differences between the two groups. Compared with non‐metformin users, patients receiving metformin tended to present with earlier disease stages, lower rates of lymph node involvement, improved treatment response, and more favorable prognosis, supporting the observed statistical findings.

### 3.8 Logistic Regression Analysis of Prognostic Factors

In the unadjusted logistic regression analysis, duration of disease, lymph node involvement, HER2 status, and metformin use were significantly associated with prognosis. In the adjusted model, duration of disease (OR = 0.784, 95% CI: 0.691–0.890, *p* < 0.001), HER2 status (OR = 0.194, 95% CI: 0.044–0.859, *p* = 0.031), and metformin use (OR = 3.34, 95% CI: 1.14–7.86, *p* = 0.026) remained independently associated with prognosis (Table 2).

**TABLE 2:** Unadjusted and adjusted logistic regression analysis of factors associated with prognosis in breast cancer patients with type 2 diabetes mellitus.

Variable	Unadjusted				Adjusted			
	*B*	Exp(B)	95% CI	*P*	*B*	Exp(B)	95% CI	*P*
Duration	−0.257	0.774	0.684–0.875	< 0.001	−0.243	0.784	0.691–0.890	< 0.001
Lymph node	−1.285	0.277	0.114–0.675	0.005				
HER2	−1.540	0.214	0.054–0.845	0.028	−1.642	0.194	0.44–0.859	0.031
Metformin	1.139	3.124	1.370–7.125	0.007	1.096	0.334	1.140–7.862	0.026

Because all patients with stage 3–4 had a poor prognosis, standard logistic regression could not be performed. Therefore, we present the results from Fisher's exact test and risk ratio instead. The association between stage and prognosis was statistically significant (Fisher's exact test, *p* < 0.001). The risk ratio for a weak prognosis in the stage 3–4 group compared to the stage = 0–1–2 group was 10.85 (95% CI: 4.98–23.63), indicating that patients with stage = 3–4 were approximately 11 times more likely to have a weak prognosis.

## 4 Discussion

The results of the present study indicate that metformin use was associated with shorter disease duration, lower disease stages, and fewer involved nodules in diabetic breast cancer patients compared to those without a history of metformin use. Metformin was associated with a better treatment response and prognosis in diabetic breast cancer patients. However, the study did not find a significant effect of metformin on the ER and HER2 status in these patients. Taken together, these findings suggest that the potential benefit of metformin may be more closely related to disease burden and clinical course rather than alterations in intrinsic tumor molecular characteristics. Additionally, the metformin group had fewer cases of recurrence and metastasis than the non‐metformin group, although the difference was not significant.

Metformin is affordable and can be used for more than just polycystic ovarian syndrome, weight loss, and blood glucose reduction. It can also protect against cardiovascular and neurodegenerative diseases [25, 26]. It is a safe drug, and the most common side effect of metformin is mild gastrointestinal discomfort, which is seen in 20%–30% of cases [27, 28]. These characteristics make it a suitable candidate for evaluation as an adjunct therapy in cancer patients, particularly in real‐world clinical settings.

Our study demonstrated that metformin users were at lower disease stages and had fewer involved lymph nodes than non‐users. These findings align with those of Basic et al., indicating that the percentage of T3 or T4 tumors was lower in metformin users than in non‐users [29]. Similarly, Min et al. reported that metformin users had fewer lymph node metastases than controls. Additionally, the luminal pattern ratio was higher in the metformin group than in the control group [24]. The consistency of these findings suggests that metformin use may be associated with less advanced disease. However, in a population‐based study by Lega et al. metformin influenced tumor stage and type of tumors in older women with diabetes who presented with breast cancer; however, after adjusting for demographic and comorbid variables, no significant association was found between metformin use and disease stage or the number of involved nodules [30]. These findings contrast with those of the present study, and the discrepancies may be due to differences in study populations (age, comorbidities), sample size, statistical adjustments, and the duration of metformin use, which could influence its effects on breast cancer outcomes.

Although experimental studies have proposed several molecular mechanisms for the anticancer effects of metformin, including pathways involving AMPK and mTOR [31, 32, 33], these mechanisms were not directly assessed in the present study. In this study, metformin use was associated with a higher treatment response rate in patients with breast cancer. In this regard, it has been shown that combining metformin with chemotherapeutic drugs such as doxorubicin, paclitaxel, and carboplatin enhances tumor regression and prevents relapse in mouse xenografts. It also enables reduced chemotherapy doses while maintaining effectiveness, particularly in targeting chemotherapy‐resistant cancer stem cells [34]. Moreover, when metformin is combined with trastuzumab, it can decrease tumor volume by more than four‐fold and help overcome primary resistance in a trastuzumab‐refractory HER2+ breast cancer model [35]. Overall, these findings suggest a potential role of metformin as an adjunct therapy in improving treatment response in breast cancer patients.

Our study did not find a significant effect of metformin on ER and HER2 status in breast cancer. Similarly, Lega et al., Min et al., and Berstein et al. reported no significant association between metformin use and ER or HER2 expression [24, 30, 36] These consistent findings across multiple studies suggest that metformin may not influence hormone receptor expression, particularly in short‐term or retrospective analyses.

In contrast, the findings of Park et al. contradict the results of the present study. Their study found that long‐term metformin use (≥ 10 years) was linked to a stronger reduction in the risk of ER‐positive breast cancer, although not statistically significant, and increased the risk of ER‐negative breast cancer and TNBC [37]. Similarly, long‐term metformin use was found to have a protective effect against ER‐positive/HER2‐negative breast cancer in a case–control study of 23 Spanish hospitals [38]. Nevertheless, Aksoy et al. reported that metformin use was associated with a higher incidence of ER‐positive breast cancer and a lower incidence of TNBC, while also being linked to better clinicopathological features [39]. These discrepancies may be due to variations in the study design, breast cancer subtypes among the study participants, patient populations, and metformin exposure duration.

Women with diabetes have higher breast cancer‐specific and all‐cause mortality [9]. In terms of prognosis, this study has elucidated that metformin use may be associated with better prognosis in patients with breast cancer. Likewise, several studies have shown a favorable effect of metformin in reducing deaths and improving prognosis in breast cancer [12, 40, 41]. Most studies have shown that metformin reduces the mortality risk of breast cancer, particularly when it is used in early‐stage breast cancer. However, since all of these studies were observational, there were variations in the severity and duration of the disease, and there was a lack of information about molecular subtypes and tumor stage, so care must be used [42].

In our study, the metformin group had fewer cases of recurrence and metastasis than the non‐metformin group, although the difference was not statistically significant. Similarly, Min et al. found a lower recurrence and metastasis rate in the metformin group, which was not statistically significant, supporting our findings [24]. The absence of statistical significance in this study and ours likely reflects the limited sample size. However, previous studies have suggested the antimetastatic effects of metformin on breast cancer [43, 44].

In terms of recurrence, other studies have also reported that metformin and weight loss modestly reduced insulin, estrogen, and androgen levels while increasing sex‐hormone binding globulin (SHBG), suggesting potential benefits in lowering breast cancer recurrence and mortality risk in overweight/obese postmenopausal survivors [45]. Moreover, while some studies have shown a protective effect of metformin against breast cancer recurrence [45, 46], others, including ours, have reported a lower but not statistically significant impact [12, 13]. Discrepancies in recurrence may be due to differences in the study design, sample size, follow‐up, patient characteristics, and confounding factors.

The retrospective, cross‐sectional nature of the study precludes causal inference and does not allow determination of whether metformin use preceded improved outcomes or whether a duration–response relationship exists. Second, detailed information regarding reasons for metformin non‐initiation and metformin exposure, including individual dose, duration of use, adherence, and timing relative to cancer diagnosis, was not available, representing a limitation given conflicting evidence on the long‐term effects of metformin. Third, the absence of multivariate analyses adjusting for potential confounders such as body mass index, glycemic control, duration of diabetes, comorbidities, and cancer treatment regimens may lead to confounding by indication. Fourth, the relatively small sample size limited the statistical power of subgroup analyses for ER and HER2 status, metastasis, and recurrence, restricting the strength of conclusions regarding molecular subtypes. Fifth, survival analyses could not be performed due to the retrospective cross‐sectional design and limited follow‐up data, restricting the ability to assess the impact of metformin on long‐term outcomes. Finally, as this study was conducted in a single center in Iran, the findings may not be generalizable to other populations. Future multicenter studies with larger sample sizes and longitudinal follow‐up are needed to confirm these findings. Cohort studies with dose–response analyses and stratification by glycemic control, as well as randomized clinical trials in diabetic breast cancer patients, would help clarify the clinical impact of metformin. Additionally, incorporating metabolic and molecular biomarkers would further elucidate its potential anticancer mechanisms and move beyond observational associations. This study did not assess standard survival endpoints such as OS, DFS, or PFS. The prognosis variable was based on retrospective clinical records and should therefore be interpreted cautiously.

## 5 Conclusion

Our study suggests that metformin use in diabetic breast cancer patients is associated with a lower disease stage, fewer involved lymph nodes, better treatment response, and improved prognosis compared with non‐users. Although no significant differences were observed in ER and HER2 status, metformin users exhibited trends toward lower recurrence and metastasis rates. The study can be positioned as providing population‐specific evidence derived from a real‐world clinical cohort in an underrepresented region, thereby adding geographic and ethnic relevance to the existing literature. These findings support the consistency of metformin's association with favorable breast cancer outcomes across diverse populations. Additionally, the use of real‐world clinical data and the exclusion of patients receiving other hypoglycemic agents allows for a cleaner comparison between metformin users and non‐users. Large‐scale prospective studies and clinical trials are needed to confirm these findings.

## Author Contributions

**Ensiyeh Bahadoran:** writing – original draft, writing – review and editing, investigation. **Yazdan Zafari:** writing – review and editing, validation, methodology. **Monir Mirzadeh:** writing – review and editing, formal analysis. **Mohsen Jafari:** writing – review and editing, methodology, formal analysis. **Sahar Moghbelinejad:** conceptualization, writing – original draft, writing – review and editing, supervision, project administration, methodology, data curation.

## Funding

The Cellular and Molecular Research Center at Qazvin University of Medical Sciences Shahid Babaei School of Medicine supported this work (grant number 401000401).

## Ethics Statement

Approval was granted by the Ethics Committee of Qazvin University of Medical Sciences IR.QUMS.REC.1402.239.

## Conflicts of Interest

The authors declare no conflicts of interest.

## Data Availability

The data that support the findings of this study are available from the corresponding author upon reasonable request.
